# Efficacy and safety of low-molecular-weight collagen peptides in knee osteoarthritis: a randomized, double-blind, placebo-controlled trial

**DOI:** 10.3389/fnut.2025.1644899

**Published:** 2025-09-04

**Authors:** Sun-Young Park, Sang-Hyun Lee, Hyun Tae Kim, Hye-Jin Park, Do-Un Kim, Seung Un Kim, In Heo

**Affiliations:** ^1^School of Korean Medicine, Pusan National University, Yangsan, Republic of Korea; ^2^Department of Korean Medicine, The Graduate School, Pusan National University, Yangsan, Republic of Korea; ^3^Health Food Research and Development, NEWTREE Co., Ltd., Seoul, Republic of Korea; ^4^EVERSPRING Co., Ltd., Seongnam, Republic of Korea; ^5^Department of Korean Medicine Rehabilitation, Pusan National University Korean Medicine Hospital, Yangsan, Republic of Korea

**Keywords:** knee osteoarthritis, randomized controlled trial, low-molecular-weight collagen peptide, nutritional supplement, joint pain relief

## Abstract

**Introduction:**

Knee osteoarthritis (OA) is a common degenerative joint disease with limited safe, long-term treatment options. Nutritional interventions, such as low-molecular-weight collagen peptides (LMCP), have emerged as promising non-pharmacological strategies for joint health. However, clinical evidence is insufficient. Therefore, we aimed to assess the efficacy and safety of LMCP supplementation in patients with knee OA.

**Methods:**

In this double-blind, randomized, placebo-controlled trial, 80 adults aged 40–75 with Kellgren–Lawrence (KL) grade I or II OA received either 3,000 mg/day LMCP or a placebo for 180 days. Primary endpoint included changes in Western Ontario and McMaster Universities Osteoarthritis (WOMAC) pain scores. Secondary endpoints included visual analog scale (VAS), WOMAC physical function and total scores, joint space width (JSW), and inflammatory markers. Adverse events (AEs) and patient compliance were monitored throughout the study.

**Results:**

LMCP supplementation significantly reduced WOMAC pain compared to the placebo (−1.90 ± 4.14 vs. 0.61 ± 3.97; *p* = 0.006). Improvements in WOMAC physical function and total scores were greater in the LMCP group than in the placebo group (−4.10 ± 9.64 vs. 0.71 ± 6.47, *p* = 0.035; −6.24 ± 14.69 vs. −0.45 ± 9.08; *p* = 0.028, respectively). No significant changes were observed in JSW or inflammatory markers. No AEs occurred.

**Conclusions:**

Daily supplementation of 3,000 mg of LMCP for 180 days was safe and effective in relieving joint pain and improving function in patients with KL grade I or II knee OA. LMCP is a promising nutrition-based non-pharmacological therapeutic option, particularly for individuals seeking complementary options to long-term non-steroidal anti-inflammatory drug therapy.

**Clinical trial registration:**

The trial was prospectively registered at the Clinical Research Information Service of South Korea (registration number: KCT0005507). The study was conducted at Pusan National University Korean Medicine Hospital (https://cris.nih.go.kr/).

## 1 Introduction

Knee osteoarthritis (OA) is the most common and clinically significant form of OA, affecting the tibiofemoral and patellofemoral compartments of the knee joint (1). It is characterized by progressive degeneration of articular cartilage, subchondral bone changes, osteophyte formation, and synovial inflammation, leading to pain, stiffness, and functional limitations. The disease predominantly affects older adults and is strongly influenced by aging, obesity, mechanical stress, and joint malalignment (2). According to the Global Burden of Disease Study, OA ranked as the seventh leading cause of disability among adults aged 70 years and older in 2021, with the number of affected individuals expected to exceed 78 million by 2040 (3, 4). Knee OA substantially impairs patients' quality of life and functional independence, increasing healthcare utilization and socioeconomic burden (5).

Pharmacologic treatments, including non-steroidal anti-inflammatory drugs (NSAIDs), are commonly used to alleviate symptoms, but their long-term use is limited by potential adverse effects such as gastrointestinal, cardiovascular, hepatic, and renal complications (6–8). These safety concerns have led to growing interest in non-pharmacologic interventions that are safer for long-term use. Recently, there has been increasing interest in nutritional strategies that can complement conventional treatments for OA by promoting cartilage health and mitigating chronic, low-grade inflammation.

Collagen supplementation has gained attention as a potential strategy for OA management, as collagen is a major structural protein in cartilage and connective tissue (9). Among various forms, low-molecular-weight collagen peptides (LMCP) produced by enzymatic hydrolysis exhibit enhanced bioavailability and contain key amino acids such as glycine, proline, and hydroxyproline that support extracellular matrix (ECM) synthesis (10–13). However, LMCPs differ in source, collagen type, and processing methods, which influence their clinical effects (10, 14). Fish-derived LMCPs, in particular, offer superior absorption due to their lower molecular weight and unique peptide profile (15).

The LMCP used in this study was derived from *Pangasius hypophthalmus* skin, rich in type I collagen, and processed via a proprietary enzymatic method (16, 17). Importantly, our preclinical studies showed that this formulation stimulated type II collagen and expression in chondrocytes and protected cartilage in an OA animal model, suggesting cartilage-specific regenerative potential beyond typical type I supplementation (18).

Despite the growing availability of LMCP-based products, there is a notable lack of randomized controlled trials evaluating their clinical efficacy in well-defined OA populations (19–22). Therefore, the present study was designed to assess the effects of daily supplementation with a specific LMCP formulation over a 180-day period in adults with Kellgren–Lawrence (KL) grade I or II knee OA, in alignment with OARSI recommendations for evaluating symptom-modifying interventions (23).

## 2 Methods and materials

### 2.1 Study design and ethical approval

This study was a randomized, double-blind, placebo-controlled clinical trial conducted at Pusan National University Korean Medicine Hospital. The study protocol was reviewed and approved by the Institutional Review Board (IRB No. 2020006) and complied with the principles of the Declaration of Helsinki and Good Clinical Practice guidelines. Written informed consent was obtained from all participants before enrollment. The trial was prospectively registered at the Clinical Research Information Service of South Korea (registration number: KCT0005507). The first patient was enrolled on 22 October 2020.

### 2.2 Participants

Eligible participants met the following criteria: (1) adults aged 40–75 years; (2) KL grade I or II in one or both knee joints on plain radiographic examination; (3) knee arthritis pain score of ≥30 mm on a visual analog scale (VAS) of 100 mm; (4) ability to engage in normal physical activity and provide written informed consent. The exclusion criteria included: (1) current treatment for clinically significant acute or chronic diseases (cardiovascular, cerebrovascular, immune, respiratory, hepatobiliary, renal and urinary, nervous, musculoskeletal, psychiatric, infectious, blood, and neoplastic diseases); (2) history of or planned artificial knee replacement surgery; (3) diagnosed with inflammatory arthritis (e.g., rheumatoid arthritis or lupus arthritis) or secondary OA owing to systemic disease; (4) gout or recurrent pseudogout; (5) infection or severe inflammation of the knee joint such as septic arthritis; (6) lower extremity fracture within the previous 3 months; (7) clinically significant hypersensitivity to collagen components; (8) uncontrolled hypertension; (9) creatinine level twice above the normal upper limit; (10) aminotransferase or alanine aminotransferase levels 2.5 times above the normal upper limit; (11) use of hyaluronic acid or steroids in the knee joint within 1 month prior to screening, systemic steroid use within 1 month prior to screening (local application and inhalation were excluded), or consumption of drugs affecting knee joint pain (including NSAIDs, glucosamine, and chondroitin sulfate) within 1 month before the first intake of the nutritional supplement for the clinical trial; (12) inability to discontinue drugs or nutritional supplements affecting knee joint pain during the test period from 1 month before the first intake of the nutritional supplement for the clinical trial; (13) participation in other clinical trials within the previous month; (14) excessive alcohol intake interfering with the test; (15) pregnancy, planned pregnancy during the trial, or lactation; (16) body mass index (BMI) ≥30 kg/m^2^; (17) other reasons deemed inappropriate by the investigator.

### 2.3 Randomization and blinding

Eligible participants were randomly assigned in a 1:1 ratio to receive either LMCP or placebo using a computer-generated block randomization scheme. An independent statistician, who was not involved in participant recruitment or outcome assessment, generated the allocation sequence. Randomization codes were sealed in sequentially numbered, opaque envelopes to ensure allocation concealment. Participants were enrolled by the research team, and group assignments were carried out by a study coordinator. The study employed a double-blind design, wherein participants, investigators, outcome assessors, and data analysts were blinded to treatment allocation until the study database was finalized.

### 2.4 Intervention

Participants in the LMCP group received three tablets, each containing 500 mg LMCP, administered orally twice daily (totaling 3,000 mg/day), for a duration of 180 days. A daily dosage of 3,000 mg was chosen based on previous studies showing clinical efficacy in knee osteoarthritis (24) and dose-dependent bioavailability in pharmacokinetic analyses (25). The LMCP tablets used in this study were manufactured by NEWTREE Co., Ltd. using collagen extracted from the skin of *Pangasius hypophthalmus*, a freshwater fish widely farmed in Southeast Asia. The skin is rich in type I collagen and has been recognized as a sustainable and bioactive source for collagen peptide production (16, 17). Enzymatic hydrolysis was carried out using proteases, followed by heat inactivation, filtration, spray drying, and sieving to produce LMCP. The active ingredient was standardized to contain over 15% total tripeptides, including at least 3% Gly-Pro-Hyp, based on preclinical studies demonstrating anti-inflammatory and cartilage-protective effects (14). The LMCP and placebo tablets, each weighing 700 mg, were manufactured to ensure consistency and quality. Placebo tablets matched the active tablets in weight, size, appearance, and taste, but contained only inert substances. Both LMCP and placebo formulations underwent comprehensive chemical component analysis (Supplementary Table S1) to verify composition and dosing precision. Participants were instructed to take the tablets after meals and to maintain a consistent schedule.

### 2.5 Study visits and clinical assessments

At the baseline visit, detailed demographic and health behavior information was collected, including age, sex, BMI, smoking status, alcohol consumption, and medical and surgical history. Follow-up visits were conducted on days 45, 90, 135, and 180. At each visit, participants underwent comprehensive clinical evaluations, including measurement of vital signs, physical examinations, anthropometric assessments, and laboratory testing. Joint space width (JSW) was assessed via X-ray at baseline, day 90, and day 180. Compliance with LMCP intake was calculated at each follow-up using tablet counts and the following formula: (number of tablets consumed/number of tablets prescribed) × 100.

### 2.6 Outcome measures

The primary outcome was the change in the Western Ontario and McMaster Universities Osteoarthritis Index (WOMAC) pain score from baseline to day 180 (26). Secondary outcomes included changes in WOMAC physical function and total scores, VAS for knee pain (27), joint stiffness (WOMAC stiffness), JSW measured via radiography, patient global assessment (PGA), and inflammatory markers [erythrocyte sedimentation rate (ESR), high-sensitivity C-reactive protein (hs-CRP)]. WOMAC and VAS were assessed at baseline and at days 45, 90, 135, and 180. Radiographs for JSW were obtained at baseline, day 90, and day 180, following standardized imaging protocols.

### 2.7 Safety monitoring

Safety was evaluated through clinical interviews and laboratory assessments at baseline and each follow-up visit. Vital signs (blood pressure, heart rate, and temperature) were measured and laboratory tests, including hematological and biochemical analyses, were performed. Adverse events (AE) were defined as any undesirable medical occurrence following intake of the study product, regardless of a causal relationship. Serious AEs were defined in accordance with International Council for Harmonization guidelines and included death, life-threatening events, hospitalization, or significant disability. All AEs were documented and reported to the IRB and study sponsor. Participants were instructed to immediately report any unusual symptoms.

### 2.8 Sample size calculation

Sample size was calculated using G^*^Power 3.1 based on the ability to detect a 10-point difference in the WOMAC total score between groups, assuming a standard deviation of 14.44 from a previous RCT (28), with a power of 80% (β = 0.2) and a two-sided alpha of 0.05. This resulted in a required sample size of ~32 participants per group. To account for a 20% dropout rate, the target enrollment was set at 40 participants per group (total *n* = 80). Although, WOMAC pain was the primary outcome; however, the WOMAC total score was used for sample size estimation owing to its broader clinical relevance and strong correlation with pain-related outcomes (26).

### 2.9 Statistical analysis

Statistical analyses were performed using SAS software version 9.2 (SAS Institute, Cary, NC, USA). The primary efficacy analysis was based on the per-protocol (PP) population, defined as participants with ≥80% compliance and no major protocol deviations. To complement this, an intention-to-treat (ITT) analysis was conducted, including all randomized participants who received at least one dose and had post-baseline efficacy data. This dual analysis ensured both ideal-condition efficacy and real-world applicability (29). Missing data were handled using the last observation carried forward (LOCF) method. Continuous variables were analyzed using independent *t*-tests or Mann–Whitney *U* tests, depending on data normality (assessed via the Shapiro–Wilk test). Paired *t*-tests or Wilcoxon signed-rank tests were used for within-group comparisons. Categorical variables were assessed using chi-square or Fisher's exact tests, as appropriate. Safety analyses included all participants who received at least one dose and completed at least one follow-up safety evaluation. Statistical significance was set at *p* < 0.05 (two-sided). Given that this was an exploratory study representing the first investigation of LMCP's effects on knee OA, no adjustments for multiple comparisons were applied.

## 3 Results

### 3.1 Study population

**Figure 1:**
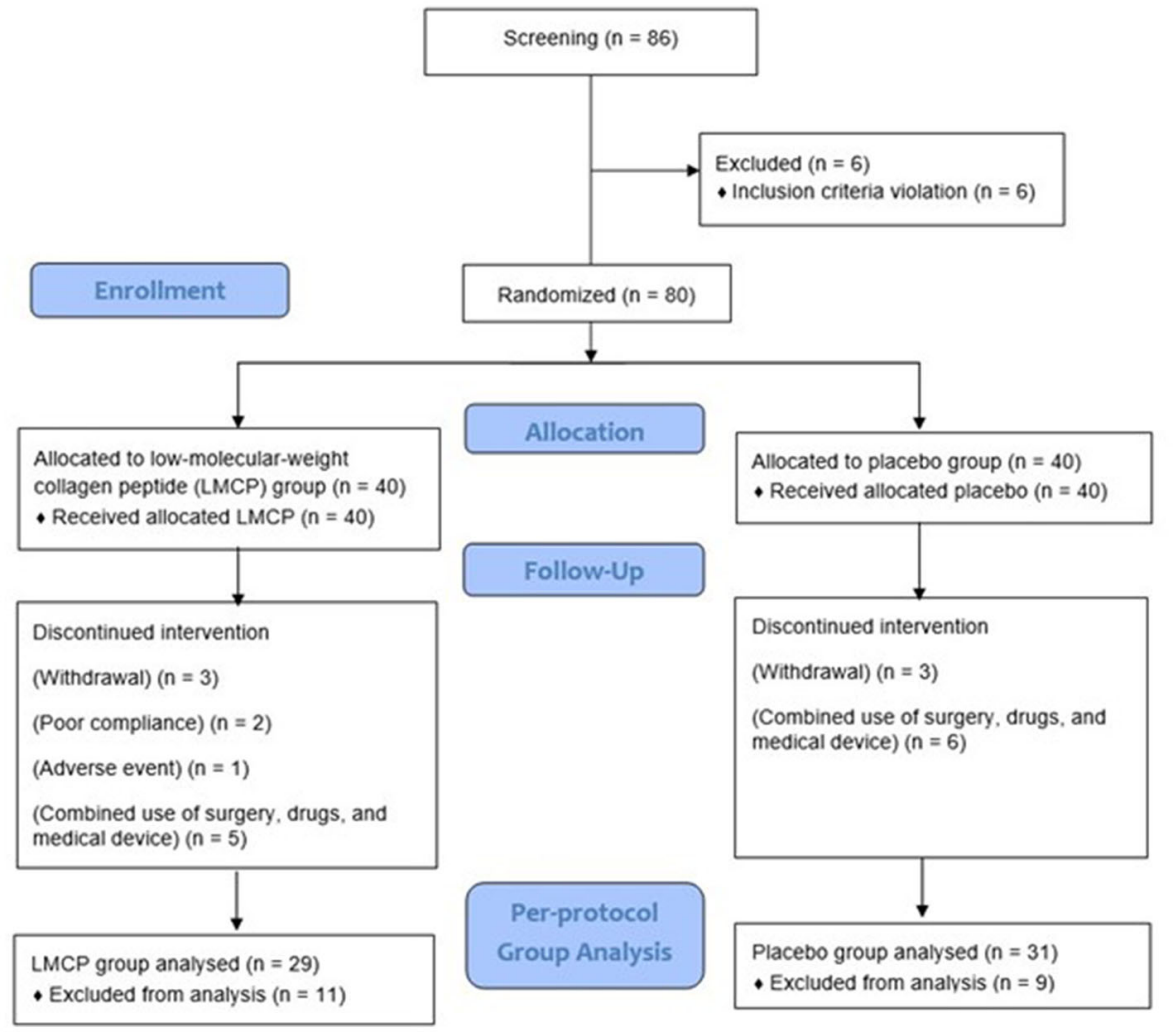
Flow chart of study participants. LMCP, low-molecular-weight collagen peptide.

A total of 86 participants were screened, and 80 were enrolled and randomized equally into LMCP (*n* = 40) and placebo (*n* = 40) groups. During the trial, six participants (three from each group) withdrew owing to personal circumstances and 11 (LMCP: five, placebo: six) dropped out for reasons including surgery, unauthorized drug use, or concurrent use of medical devices. Additionally, one participant dropped out because of AEs and two were excluded because they failed to meet the 80% criterion for medication compliance in the LMCP group. These dropouts were not related to trial progress, efficacy, or safety. Ultimately, 60 participants (LMCP: 29, placebo: 31) who completed the trial were included in the PP analysis group and contributed to the efficacy analysis (Figure 1). The safety analysis included 80 participants (LMCP: 40, placebo: 40) who received at least one dose of their assigned tablets. Baseline demographic and clinical characteristics were similar between groups, except for differences in KL grades within the PP population. No significant differences were observed in age, sex, BMI, smoking, alcohol consumption, or medication compliance. Mean medication compliance was high in both groups: 97.4 ± 3.0 (placebo) and 95.7 ± 4.4 (LMCP; Table 1).

**Table 1:** Baseline sociodemographic characteristics of adults with knee OA by assigned group.

**Variable**	**Placebo (*n* = 31)**	**LMCP (*n* = 29)**	***p* value**
Age (years)	53.81 ± 9.50	52.10 ± 7.30	0.463
Female (%)	28 (90.3)	27 (93.1)	0.750
BMI (kg/m^2^)	23.77 ± 2.74	24.11 ± 2.71	0.631
KL grade 1	24 (77.4)	15 (51.7)	0.037
KL grade 2	7 (22.6)	14 (48.3)	0.463
Current smoker	1 (3.2)	2 (6.9)	0.606
Current drinker	7 (22.6)	8 (27.6)	0.655
Medication compliance	97.42 ± 3.01	95.72 ± 4.37	0.089

Values are expressed as n (%) for dichotomous variables and as the mean ± standard deviation for continuous variables. A χ^2^ test or Fisher exact test was used for categorical variables; for continuous variables, an independent t-test or Mann–Whitney U test was used.

OA, osteoarthritis; BMI, body mass index; KL, Kellgren–Lawrence.

No adjustments for Type I errors have been made in this table.

### 3.2 Efficacy outcomes

Table 2 presents the primary and secondary efficacy outcomes, indicating the changes from baseline to day 180. Table 3 provides exploratory findings of the WOMAC subscale scores, showing changes from baseline across multiple time points.

**Table 2:** Changes in outcome measurements at baseline and 180-day follow-up in adults with knee OA.

**Variable**	**Placebo (*n* = 31)**	*p* value^*^	**LMCP (*n* = 29)**	*p* value^*^	*p* value^**^
**WOMAC (Pain)**					
Baseline	3.29 ± 2.53		4.45 ± 3.56		0.259
180-day	3.90 ± 3.88		2.55 ± 2.84		0.068
Change from baseline	0.61 ± 3.97	0.570	−1.90 ± 4.14	0.004	0.006
**VAS**					
Baseline	46.03 ± 13.40		43.93 ± 8.30		0.953
180-day	24.26 ± 16.73		26.66 ± 17.65		0.609
Change from baseline	−21.77 ± 17.83	0.001	−17.28 ± 15.18	0.001	0.299
**WOMAC (Joint stiffness)**					
Baseline	2.13 ± 1.23		2.00 ± 1.73		0.486
180-day	2.06 ± 1.69		1.59 ± 1.59		0.233
Change from baseline	−0.06 ± 1.46	0.803	−0.41 ± 1.88	0.303	0.423
**WOMAC (Physical function)**					
Baseline	12.42 ± 9.34		13.28 ± 9.41		0.667
180-day	11.71 ± 10.40		9.17 ± 9.42		0.382
Change from baseline	−0.71 ± 6.47	0.258	−4.10 ± 9.64	0.002	0.035
**WOMAC (Total)**					
Baseline	17.77 ± 12.65		19.34 ± 13.85		0.668
180-day	17.32 ± 13.81		13.10 ± 13.18		0.219
Change from baseline	−0.45 ± 9.08	0.475	−6.24 ± 14.69	0.002	0.028
**JSW (Rt.)**					
Baseline (90-day)	7.96 ± 0.93		8.26 ± 0.93		0.228
180-day	8.47 ± 0.88		8.60 ± 1.22		0.653
Change from baseline	0.51 ± 0.88		0.34 ± 1.22		
**JSW (Lt.)**					
Baseline (90-day)	7.87 ± 0.94		8.22 ± 0.90		0.168
180-day	8.50 ± 0.93		8.57 ± 0.95		0.786
Change from baseline	0.63 ± 0.93		0.35 ± 0.95		
**PGA**					
Baseline	42.90 ± 15.52		44.48 ± 17.23		0.710
180-day	43.45 ± 19.51		35.10 ± 17.90		0.090
Change from baseline	0.55 ± 18.70	0.871	−9.38 ± 20.32	0.019	0.054
**ESR**					
Baseline	8.42 ± 6.83		8.97 ± 7.10		0.640
180-day	8.65 ± 7.64		8.41 ± 5.72		0.761
Change from baseline	0.23 ± 5.46	0.889	−0.55 ± 6.70	0.654	0.624
**hs-CRP**					
Baseline	2.73 ± 11.99		0.86 ± 0.82		0.258
180-day	1.38 ± 2.09		0.81 ± 0.79		0.721
Change from baseline	−1.35 ± 11.32	0.042	−0.05 ± 1.15	0.904	0.148

Values are expressed as the mean ± standard deviation.

* Variables within group were compared using paired t-tests.

** Variables between the two groups were compared using independent t-tests or Wilcoxon rank-sum tests.

OA, osteoarthritis; LMCP, low-molecular-weight collagen peptide; WOMAC, Western Ontario and McMaster Universities Osteoarthritis; VAS, visual analog scale; JSW, joint space width; Rt., right; Lt., left; PGA, patient global assessment; ESR, erythrocyte sedimentation rate; hs-CRP, high-sensitivity C-reactive protein.

**Table 3:** Changes in WOMAC scores from baseline to days 45, 90, 135, and 180.

**Variable**	**Placebo (*n* = 31)**	*p-*value^*^	**LMCP (*n* = 29)**	*p*-value^*^	*p*-value^**^
**WOMAC (Pain)**					
Baseline	3.29 ± 2.53		4.45 ± 3.56		0.025
45-day	0.29 ± 2.18	0.528	−1.45 ± 3.31	0.053	0.099
90-day	0.45 ± 2.55	0.517	−1.28 ± 3.87	0.056	0.046
135-day	0.23 ± 2.50	0.831	−1.62 ± 3.71	0.029	0.060
180-day	0.61 ± 3.97	0.570	−1.90 ± 4.14	0.004	0.006
**WOMAC (Joint stiffness)**					
Baseline	2.13 ± 1.23		2.00 ± 1.73		0.486
45-day	0.19 ± 1.45	0.589	0.07 ± 1.83	0.608	0.849
90-day	0.00 ± 1.39	0.925	−0.14 ± 1.79	0.713	0.946
135-day	−0.06 ± 1.73	0.861	−0.28 ± 1.67	0.445	0.970
180-day	−0.06 ± 1.46	0.803	−0.41 ± 1.88	0.303	0.423
**WOMAC (Physical function)**					
Baseline	12.42 ± 9.34		13.28 ± 9.41		0.667
45-day	0.61 ± 7.87	0.680	−2.00 ± 9.43	0.352	0.870
90-day	0.26 ± 7.24	0.791	−2.38 ± 10.01	0.130	0.245
135-day	1.29 ± 10.03	0.897	−4.97 ± 8.79	0.005	0.031
180-day	−0.71 ± 6.47	0.258	−4.10 ± 9.64	0.002	0.035
**WOMAC (Total)**					
Baseline	17.77 ± 12.65		19.34 ± 13.85		0.668
45-day	0.87 ± 10.55	0.630	−3.24 ± 13.90	0.369	0.711
90-day	0.61 ± 10.56	0.982	−3.41 ± 14.91	0.194	0.230
135-day	1.39 ± 13.13	0.933	−6.48 ± 12.97	0.015	0.074
180-day	−0.45 ± 9.08	0.475	−6.24 ± 14.69	0.002	0.028

Values are expressed as the mean ± standard deviation.

* Variables within group were compared using paired t-tests.

** The variables between the two groups were compared using independent t-tests or Wilcoxon rank-sum tests.

WOMAC, Western Ontario and McMaster Universities Osteoarthritis; OA, Osteoarthritis; LMCP, Low-molecular-weight collagen peptide.

**Figure 2:**
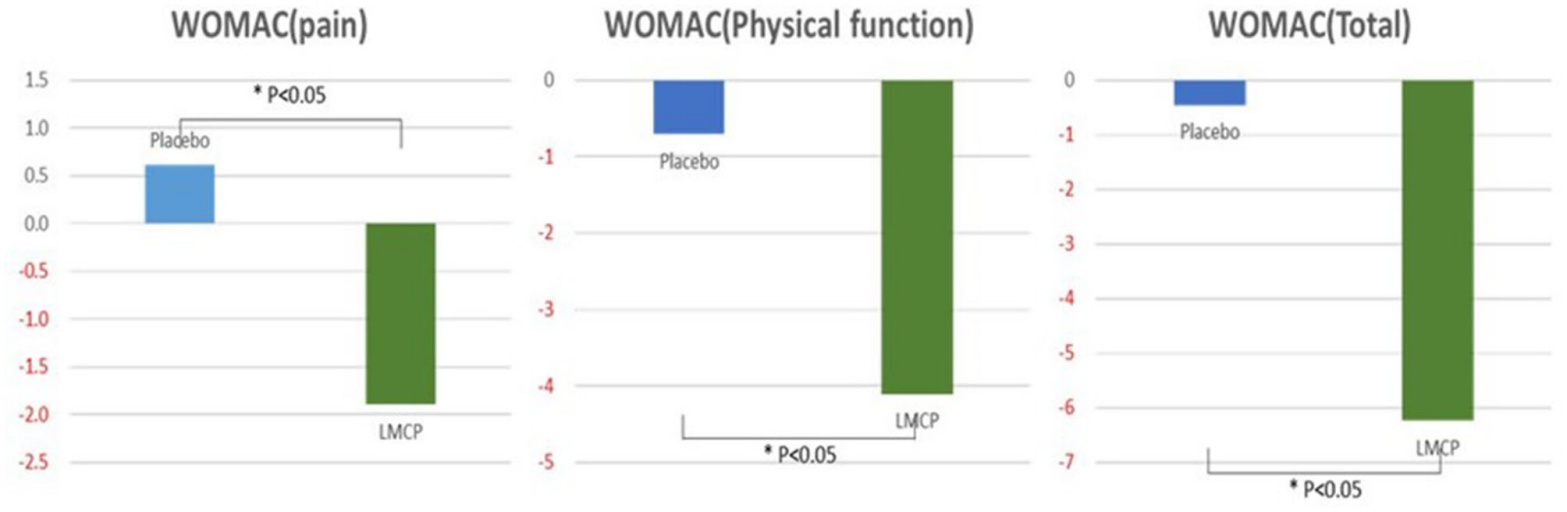
Changes in WOMAC scores from the baseline between the LMCP and placebo groups at day 180. *p < 0.05 compared with the placebo group. WOMAC, Western Ontario and McMaster Universities Osteoarthritis Index; LMCP, low-molecular-weight collagen peptide.

From baseline to day 180, the LMCP group demonstrated significantly greater improvements in WOMAC pain scores compared to the placebo group (−1.90 ± 4.14 vs. 0.61 ± 3.97; *t* = 2.395, *p* = 0.006). Similarly, from baseline to day 180, the LMCP group showed significant improvements in physical function (−4.10 ± 9.64 vs. −0.71 ± 6.47; *t* = 1.611, *p* = 0.035) and total WOMAC scores (−6.24 ± 14.69 vs. −0.45 ± 9.08; *t* = 1.850, *p* = 0.028). Both groups exhibited reductions in VAS scores from baseline; however, the between-group difference was not statistically significant (−17.28 ± 15.18 vs. −21.77 ± 17.83; *t* = −1.049, *p* = 0.299). WOMAC stiffness scores showed a greater improvement in the LMCP group but the difference between groups was not statistically significant (−0.41 ± 1.88 vs. −0.06 ± 1.46, *t* = 0.807, *p* = 0.423). Changes in inflammatory markers, including ESR (0.23 ± 5.46 vs. −0.55 ± 6.70, *t* = 0.494, *p* = 0.624) and hs-CRP (−1.35 ± 11.32 vs. −0.05 ± 1.15, *t* = −0.612, *p* = 0.904), were also not significantly different between groups. Both groups remained within the normal range for hs-CRP levels at day 180. A trend, toward improvement was observed in PGA scores in the LMCP group, although the difference was not statistically significant (−9.38 ± 20.32 vs. 0.55 ± 18.70; *t* = 1.971, *p* = 0.054; Table 2, Figure 2).

**Figure 3:**
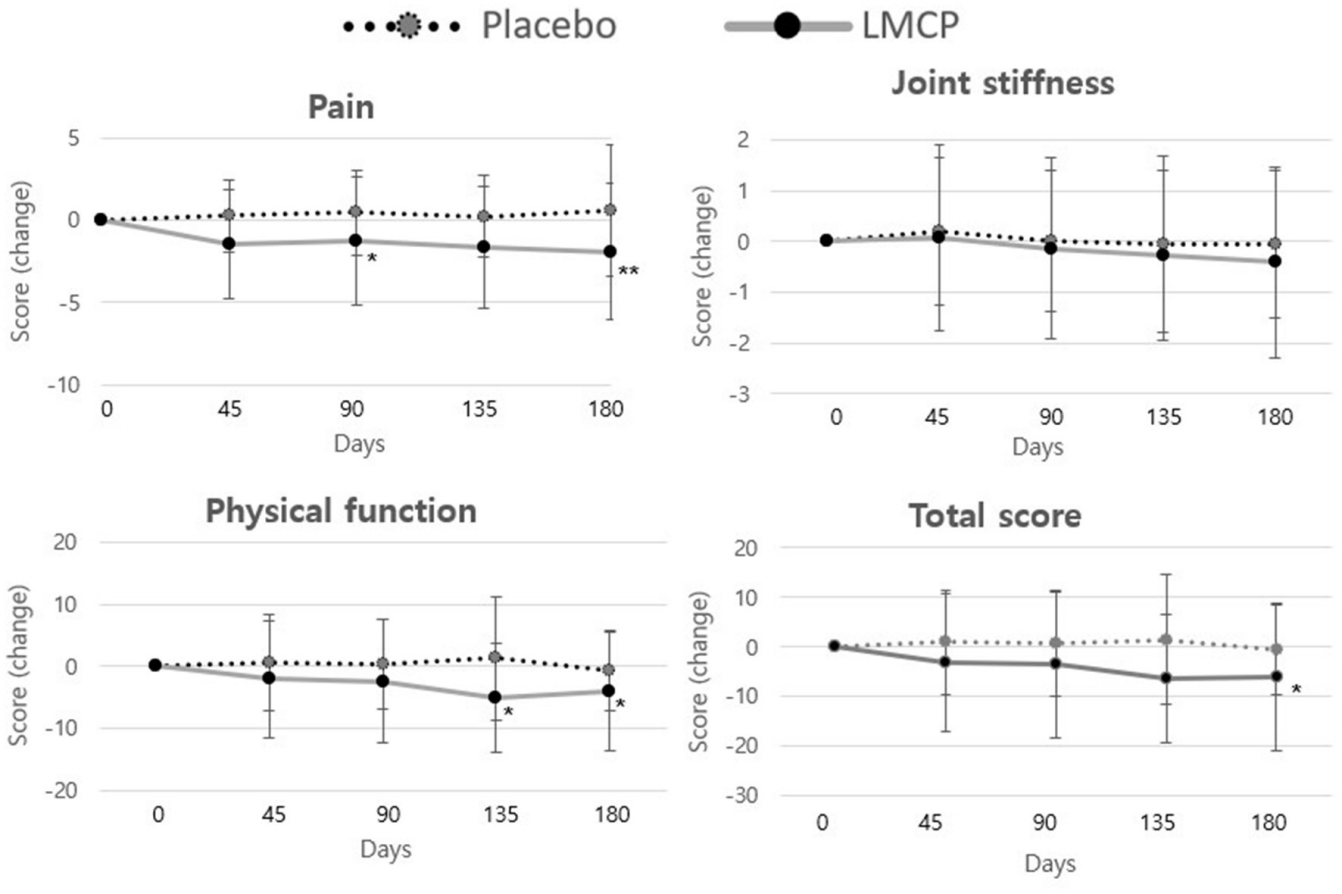
Changes in WOMAC score from baseline between the LMCP and placebo groups at days 45, 90, 135, and 180. *p < 0.05, **p < 0.01 compared with the placebo group. WOMAC, Western Ontario and McMaster Universities Osteoarthritis Index; LMCP, low-molecular-weight collagen peptide.

Longitudinal analysis of WOMAC subscale scores over time (Table 3, Figure 3) revealed that the LMCP group exhibited significantly greater improvement in pain scores on day 90 (−1.28 ± 3.87 vs. 0.45 ± 2.55; *t* = 2.053, *p* = 0.046) and in physical function on day 135 (−4.97 ± 8.79 vs. 1.29 ± 10.03; *t* = 2.562, *p* = 0.031). Stiffness scores showed minimal changes over time with no significant differences observed between groups. The results of the intention-to-treat (ITT) analysis were consistent with those from the PP analysis and are presented in Supplementary Table S2.

### 3.3 Safety

Safety assessments were performed during the trial for all participants who used the LMCP at least once. A single AE (urticaria) was determined to be unrelated to the trial and resolved without complications or sequelae. No serious AEs were reported. Laboratory parameters, vital signs, and clinical chemistry remained within normal limits and showed no significant differences between groups (Supplementary Table S3).

## 4 Discussion

This study demonstrated that daily nutritional supplementation with LMCP for 180 days effectively improved joint pain and physical function in patients with KL grade I or II knee OA. The clinical benefits observed may be partially explained by LMCP's bioactive properties, including stimulation of chondrocyte activity and promotion of ECM synthesis, as suggested by preclinical studies (15). However, these mechanisms remain speculative, as our study did not directly assess ECM-related biomarkers or tissue-level activity of the peptides. LMCPs are rich in amino acids such as glycine, proline, and hydroxyproline, which are essential for the synthesis of type II collagen, proteoglycans, and hyaluronic acid, key components in maintaining cartilage health (18, 30). In addition, their Gly–Pro–Hyp tripeptide sequence and low molecular weight (< 10 kDa) contribute to enhanced gastrointestinal absorption and systemic bioavailability, reinforcing their potential as a clinically useful nutritional ingredient (30–32).

Our findings align with those of previous reports on the efficacy of collagen peptide supplementation in OA, reinforcing LMCP's role as a viable nutrition-based non-pharmacologic intervention (19, 20). Improvements in WOMAC pain and physical function were evident by day 135 and persisted through day 180. Notably, no serious adverse events were reported, confirming that LMCP was well-tolerated over the 6-month intervention period. This favorable safety profile is particularly relevant for patients with contraindications to long-term NSAID use.

Notably, the primary outcome of WOMAC pain demonstrated a clinically meaningful improvement, with a standardized mean difference of 0.46—exceeding the minimum clinically important difference (MCID) threshold of 0.39 proposed by the American Academy of Orthopedic Surgeons (33, 34). This degree of change reinforces the potential clinical relevance of LMCP as a safe and conservative nutrition-based strategy for managing pain in patients with knee OA, particularly for those seeking to avoid or minimize long-term pharmacologic interventions.

This study has some limitations. Due to the PP analysis, 60 participants were included instead of the planned 64, but the primary outcomes remained significant (35). Future studies with adequate sample sizes are warranted to validate and extend these findings. The minimal improvement observed in joint stiffness—despite notable reductions in pain and physical function—suggests that stiffness may respond more slowly to intervention or involve distinct pathophysiologic mechanisms. Longer follow-up durations and stiffness-specific metrics are recommended to assess therapeutic effects more accurately. The absence of observable structural improvements in cartilage may be attributed to the limited 180-day observation period. Cartilage regeneration is typically a slow process requiring longer durations to manifest detectable changes. Moreover, JSW, though practical, may lack the sensitivity to detect subtle early-stage structural alterations, especially in KL grade I–II OA. Future trials should incorporate advanced imaging modalities such as magnetic resonance imaging and extend follow-up to 1–5 years for more accurate structural assessment (36, 37). The lack of significant changes in inflammatory markers, including hs-CRP, may also be related to the relatively short observation period. Systemic inflammatory modulation often requires prolonged intervention before meaningful biochemical changes are evident. Thus, longer-term studies are warranted to evaluate the biochemical impact of LMCP on inflammation more effectively (38–40). A placebo-controlled design is appropriate for exploratory trials; however, future studies should include active comparators such as NSAIDs, physiotherapy, structured exercise programs, or widely used supplements. Such comparisons would help contextualize LMCP's clinical relevance within the broader spectrum of OA management strategies (41–43). Although compliance with the six-tablet daily regimen was high—likely reflecting participants' motivation to manage symptoms—future formulations such as higher-concentration tablets or alternative dosage forms may further improve real-world adherence (44, 45). Finally, this was a single-center study involving a relatively homogenous population, which may limit the generalizability of the findings (46). Like other nutritional interventions, LMCP also requires validation across broader populations, and its potential should be interpreted within the wider context of OA management (46).

## 5 Conclusions

This randomized, placebo-controlled trial demonstrated that daily supplementation with 3,000 mg of LMCP for 180 days significantly reduced joint pain and improved physical function in patients with KL grade I or II knee OA, with no serious AEs. The magnitude of pain reduction exceeded the MCID, supporting the clinical relevance of LMCP as a safe and non-pharmacological intervention for patients with knee OA seeking complementary options to long-term NSAID use. Structural and inflammatory markers did not change significantly over the study period; however, these findings support the symptomatic benefits of LMCP as a functional nutritional approach to joint care. Future studies should incorporate longer follow-up durations, active comparators, and advanced imaging or biochemical markers to clarify the long-term role of LMCP in comprehensive, nutrition-integrated OA management.

## Data Availability

The raw data supporting the conclusions of this article will be made available by the authors, without undue reservation.

## References

[1] Neogi T. The epidemiology and impact of pain in osteoarthritis. Osteoarthritis Cartilage. (2013) 21:1145–53. 10.1016/j.joca.2013.03.018 23973124 PMC3753584

[2] Lane NE Brandt K Hawker G Peeva E Schreyer E Tsuji W. OARSI-FDA initiative: defining the disease state of osteoarthritis. Osteoarthritis Cartilage. (2011) 19:478–82. 10.1016/j.joca.2010.09.013 21396464

[3] GBD 2021 Osteoarthritis Collaborators. Global, regional, and national burden of osteoarthritis, 1990–2020 and projections to 2050: a systematic analysis for the Global Burden of Disease Study 2021. Lancet Rheumatol. (2023) 5:e508–22. 10.1016/S2665-9913(23)00163-7 37675071 PMC10477960

[4] Barbour KE Helmick CG Boring M Brady TJ. Vital signs: prevalence of doctor-diagnosed arthritis and arthritis-attributable activity limitation—United States, 2013–2015. MMWR Morb Mortal Wkly Rep. (2017) 66:246–53. 10.15585/mmwr.mm6609e1 28278145 PMC5687192

[5] Sampoorna M Mahender M Bhavani SV. Ortholord tablets: a blend of natural ingredients provides nutritional support for joint health. Asian J Appl Sci Technol. (2020) 4:17–36. 10.38177/AJAST.2020.4204

[6] Machado GC Maher CG Ferreira PH Pinheiro MB Lin CW Day RO. Efficacy and safety of paracetamol for spinal pain and osteoarthritis: systematic review and meta-analysis of randomised placebo controlled trials. BMJ. (2015) 350:h1225. 10.1136/bmj.h1225 25828856 PMC4381278

[7] McAlindon TE Bannuru RR Sullivan MC Arden NK Berenbaum F Bierma-Zeinstra SM. OARSI guidelines for the non-surgical management of knee osteoarthritis. Osteoarthritis Cartilage. (2014) 22:363–88. 10.1016/j.joca.2014.01.003 24462672

[8] Roberts E Delgado Nunes V Buckner S Latchem S Constanti M Miller P. Paracetamol: not as safe as we thought? A systematic literature review of observational studies. Ann Rheum Dis. (2016) 75:552–9. 10.1136/annrheumdis-2014-206914 25732175 PMC4789700

[9] Lopez HL. Nutritional interventions to prevent and treat osteoarthritis. Part II: focus on micronutrients and supportive nutraceuticals. PM R. (2012) 4:S155–68. 10.1016/j.pmrj.2012.02.023 22632695

[10] Yamamoto S Hayasaka F Deguchi K Okudera T Furusawa T Sakai Y. Absorption and plasma kinetics of collagen tripeptide after peroral or intraperitoneal administration in rats. Biosci Biotechnol Biochem. (2015) 79:2026–33. 10.1080/09168451.2015.1062711 26155906

[11] Amigo L Hernández-Ledesma B. Current evidence on the bioavailability of food bioactive peptides. Molecules. (2020) 25:4479. 10.3390/molecules25194479 33003506 PMC7582556

[12] Kumar S Sugihara F Suzuki K Inoue N Venkateswarathirukumara S. A double-blind, placebo-controlled, randomised, clinical study on the effectiveness of collagen peptide on osteoarthritis. J Sci Food Agric. (2015) 95:702–7. 10.1002/jsfa.6752 24852756

[13] Tsuruoka N Yamato R Sakai Y Yoshitake Y Yonekura H. Promotion by collagen tripeptide of type I collagen gene expression in human osteoblastic cells and fracture healing of rat femur. Biosci Biotechnol Biochem. (2007) 71:2680–7. 10.1271/bbb.70287 17986775

[14] Zague V de Freitas V da Costa Rosa M Oliveira N de Souza M Maia Campos P. Collagen hydrolysate intake increases skin collagen expression and suppresses matrix metalloproteinase 2 activity. J Med Food. (2011) 14:618–24. 10.1089/jmf.2010.0085 21480801

[15] Bello AE Oesser S. Collagen hydrolysate for the treatment of osteoarthritis and other joint disorders: a review of the literature. Curr Med Res Opin. (2006) 22:2221–32. 10.1185/030079906X148373 17076983

[16] Gustami VA Nurilmala M Jacoeb AM. Characteristics of Collagen from Swim Bladder of Catfish (Pangasius sp.) Under Different Ultrasound Exposure Times. BIO Web of Conferences 147, 01023 (2024). 10.1051/bioconf/202414701023

[17] Kim SI Park SH Na W Shin YC Oh MS Sim YE. Dietary collagen hydrolysates retard estrogen deficiency-induced bone loss through blocking osteoclastic activation and enhancing osteoblastic matrix mineralization. Biomedicines. (2022) 10:1382. 10.3390/biomedicines10061382 35740404 PMC9219917

[18] Lee MH Kim HM Chung HC Kim DU Lee JH. Low-molecular-weight collagen peptide ameliorates osteoarthritis progression through promoting extracellular matrix synthesis by chondrocytes in a rabbit anterior cruciate ligament transection model. J Microbiol Biotechnol. (2021) 31:1401–8. 10.4014/jmb.2108.08027 34528913 PMC9705828

[19] Kumar P Bansal P Rajnish RK Sharma S Dhillon MS Patel S. Efficacy of undenatured collagen in knee osteoarthritis: review of the literature with limited meta-analysis. Am J Transl Res. (2023) 15:5545–55. 37854210 PMC10579002

[20] Bakilan F Armagan O Ozgen M Tascioglu F Bolluk O Alatas O. Effects of native type II collagen treatment on knee osteoarthritis: a randomized controlled trial. Eurasian J Med. (2016) 48:95–101. 10.5152/eurasianjmed.2015.15030 27551171 PMC4970562

[21] Lugo JP Saiyed ZM Lane NE. Efficacy and tolerability of an undenatured type II collagen supplement in modulating knee osteoarthritis symptoms: a multicenter randomized, double-blind, placebo-controlled study. Nutr J. (2016) 15:14. 10.1186/s12937-016-0130-8 26822714 PMC4731911

[22] Liu X Machado GC Eyles JP Ravi V Hunter DJ. Dietary supplements for treating osteoarthritis: a systematic review and meta-analysis. Br J Sports Med. (2018) 52:167–75. 10.1136/bjsports-2016-097333 29018060

[23] Pham T Van Der Heijde D Lassere M Altman RD Anderson JJ Bellamy N. Outcome variables for osteoarthritis clinical trials: the OMERACT-OARSI set of responder criteria. J Rheumatol. (2003) 30:1648–54. 12858473

[24] Yamamoto S Masuda K Saitou M Maruyama K Sakai Y. Effects of collagen tripeptide on knee osteoarthritis in humans and animals. Pharmacometrics. (2015) 89:115–24.

[25] Yamamoto S Deguchi K Onuma M Numata N Sakai Y. Absorption and urinary excretion of peptides after collagen tripeptide ingestion in humans. Biol Pharm Bull. (2016) 39:428–34. 10.1248/bpb.b15-00624 26934933

[26] McConnell S Kolopack P Davis AM. The Western Ontario and McMaster Universities Osteoarthritis Index (WOMAC): a review of its utility and measurement properties. Arthritis Rheum. (2001) 45:453–61. 10.1002/1529-0131(200110)45:5<453::AID-ART365>3.0.CO;2-W 11642645

[27] Bassuk SS Rifai N Ridker PM. High-sensitivity C-reactive protein: clinical importance. Curr Probl Cardiol. (2004) 29:439–93. 10.1016/S0146-2806(04)00074-X 15258556

[28] Park SH Kim SK Shin IH Kim HG Choe JY. Effects of AIF on knee osteoarthritis patients: double-blind, randomized placebo-controlled study. Korean J Physiol Pharmacol. (2009) 13:33–7. 10.4196/kjpp.2009.13.1.33 19885024 PMC2766718

[29] Ranganathan P Pramesh CS Aggarwal R. Common pitfalls in statistical analysis: intention-to-treat versus per-protocol analysis. Perspect Clin Res. (2016) 7:144–6. 10.4103/2229-3485.184823 27453832 PMC4936074

[30] Maldonado M Nam J. The role of changes in extracellular matrix of cartilage in the presence of inflammation on the pathology of osteoarthritis. Biomed Res Int. (2013) 2013:284873. 10.1155/2013/284873 24069595 PMC3771246

[31] Inerot S Heinegård D Audell L Olsson SE. Articular-cartilage proteoglycans in aging and osteoarthritis. Biochem J. (1978) 169:143–56. 10.1042/bj1690143 629741 PMC1184203

[32] Ng KW Saliman JD Lin EY Statman LY Kugler LE Lo SB. Culture duration modulates collagen hydrolysate-induced tissue remodeling in chondrocyte-seeded agarose hydrogels. Ann Biomed Eng. (2007) 35:1914–23. 10.1007/s10439-007-9373-z 17721729

[33] Concoff A Rosen J Fu F Bhandari M Boyer K Karlsson J. A comparison of treatment effects for nonsurgical therapies and the minimum clinically important difference in knee osteoarthritis: a systematic review. JBJS Rev. (2019) 7:e5. 10.2106/JBJS.RVW.18.00150 31415278 PMC6727942

[34] Lee SH Kao CC Liang HW Wu HT. Validity of the Osteoarthritis Research Society International (OARSI) recommended performance-based tests of physical function in individuals with symptomatic Kellgren and Lawrence grade 0–2 knee osteoarthritis. BMC Musculoskelet Disord. (2022) 23:1040. 10.1186/s12891-022-06012-2 36451167 PMC9714223

[35] Bacchetti P. Current sample size conventions: flaws, harms, and alternatives. BMC Med. (2010) 8:17. 10.1186/1741-7015-8-17 20307281 PMC2856520

[36] Vignon E Piperno M Le Graverand MP Mazzuca SA Brandt KD Mathieu P. Measurement of radiographic joint space width in the tibiofemoral compartment of the osteoarthritic knee: comparison of standing anteroposterior and Lyon schuss views. Arthritis Rheum. (2003) 48:378–84. 10.1002/art.10773 12571846

[37] Menashe L Hirko K Losina E Kloppenburg M Zhang W Li L. The diagnostic performance of MRI in osteoarthritis: a systematic review and meta-analysis. Osteoarthritis Cartilage. (2012) 20:13–21. 10.1016/j.joca.2011.10.003 22044841 PMC3934362

[38] Pearle AD Scanzello CR George S Mandl LA DiCarlo EF Peterson M. Elevated high-sensitivity C-reactive protein levels are associated with local inflammatory findings in patients with osteoarthritis. Osteoarthritis Cartilage. (2007) 15:516–23. 10.1016/j.joca.2006.10.010 17157039

[39] Dias IR Viegas CA Carvalho PP. Large animal models for osteochondral regeneration. Adv Exp Med Biol. (2018) 1059:441–501. 10.1007/978-3-319-76735-2_20 29736586

[40] Elshahid ZA Salama A Gouhar SA. Assessment of the synergistic anti-inflammatory effect of naringin/sulindac for the treatment of osteoarthritis: *in vitro* and *in vivo*. Adv Tradit Med. (2024) 24:265–83. 10.1007/s13596-023-00692-4

[41] Orsso CE Ford KL Kiss N Trujillo EB Spees CK Hamilton-Reeves JM. Optimizing clinical nutrition research: the role of adaptive and pragmatic trials. Eur J Clin Nutr. (2023) 77:1130–42. 10.1038/s41430-023-01330-7 37715007 PMC10861156

[42] Staudacher HM Irving PM Lomer MCE Whelan K. The challenges of control groups, placebos and blinding in clinical trials of dietary interventions. Proc Nutr Soc. (2017) 76:203–12. 10.1017/S0029665117000350 28629483

[43] Patsopoulos NA. A pragmatic view on pragmatic trials. Dialogues Clin Neurosci. (2011) 13:217–24. 10.31887/DCNS.2011.13.2/npatsopoulos 21842619 PMC3181997

[44] Uršulin-Trstenjak N Poljak D Šarkanj B Sajko M Šarkanj ID. The impact of education sources on patient compliance with the recommended oral nutritional supplement (ONS) intake: a qualitative survey. Nutrients. (2025) 17:889. 10.3390/nu17050889 40077759 PMC11901901

[45] Hubbard GP Elia M Holdoway A Stratton RJ. A systematic review of compliance to oral nutritional supplements. Clin Nutr. (2012) 31:293–312. 10.1016/j.clnu.2011.11.020 22257636

[46] Chung KC Song JW WRIST Study Group. A guide to organizing a multicenter clinical trial. Plast Reconstr Surg. (2010) 126:515–23. 10.1097/PRS.0b013e3181df64fa 20375760 PMC2917608

