# Supplementary material for: Efficacy and safety of low-molecular-weight collagen peptides in knee osteoarthritis: a randomized, double-blind, placebo-controlled trial
Source: Front Nutr. 2025 Sep 4;12:1644899. doi: 10.3389/fnut.2025.1644899 (PMC12445226; doi:10.3389/fnut.2025.1644899)
Supplement: Supplementary file 1 [file Table_1.docx]

Supplementary Material

# Supplementary Tables

**Table S1.** List of chemical components in the tablets for the LMCP and placebo groups

| **Ingredients** | | **Contents** | |
| --- | --- | --- | --- |
|  |  | **LMCP (n=40)** **(mg)** | **Placebo (n=40)** **(mg)** |
| Low-molecular-weight Collagen peptide | | 500 | 0 |
| Indigestible maltodextrin | | 0 | 259.00 |
| Crystalline cellulose | | 94.625 | 173.53 |
| Dextrin | | 70 | 270.10 |
| Carboxymethylcellulose Sodium | | 14 | 14.80 |
| Silicon dioxide  Magnesium Stearate |  | 10.5 | 11.10 |
|  |  | 7 | 7.40 |
| Droxypropylmethylcellulose | | 3.5 | 3.70 |
| Glycerin Fatty Acid Esters | | 0.375 | 0.37 |
| **Total** | | 700.000 | 740.000 |

LMCP, Low-molecular collagen peptides.

**Table S2.** Changes in outcome measurements between baseline and day 180 among participants in the ITT group

| **Variable** | **Baseline** | | | |  | | **180 days Change from baseline** | | |
| --- | --- | --- | --- | --- | --- | --- | --- | --- | --- |
|  | **Placebo (n=40)** | **LMCP (n=40)** | **p**** | **Placebo (n=40)** | | **p*** | **LMCP (n=40)** | **p*** | **p**** |
| **WOMAC (Pain)** | 4.13±3.10 | 4.43±3.33 | 0.720 | -0.30±4.29 | | 0.315 | -1.20±3.74 | 0.025 | 0.386 |
| **VAS** | 46.95±13.70 | 45.03±9.03 | 0.919 | -20.55±19.81 | | <0.001 | -14.40±16.61 | <0.001 | 0.136 |
| **JSW**^***^ **(Rt.)** | 7.93±0.88 | 8.39±1.00 | 0.052 | 0.42±0.89 | |  | 0.21±1.22 |  | 0.653 |
| **JSW**^***^ **(Lt.)** | 7.77±0.93 | 8.29±0.92 | 0.027 | 0.73±0.93 | |  | 0.28±0.95 |  | 0.786 |
| **WOMAC**  **(Joint stiffness)** | 2.30±1.38 | 2.08±1.54 | 0.470 | -0.28±1.54 | | 0.298 | -0.33±1.72 | 0.321 | 0.984 |
| **WOMAC**  **(Physical function)** | 14.98±11.24 | 13.85±9.05 | 0.897 | -2.60±9.14 | | 0.045 | -3.20±8.54 | 0.001 | 0.436 |
| **WOMAC (Total)** | 21.23±15.05 | 20.18±13.14 | 0.939 | -3.28±12.58 | | 0.066 | -4.70±13.06 | 0.003 | 0.443 |
| **PGA** | 44.85±15.50 | 43.60±17.60 | 0.737 | 2.05±17.67 | | 0.467 | -7.83±19.02 | 0.013 | 0.018 |
| **ESR** | 7.73±6.28 | 9.82±6.90 | 0.089 | 0.05±4.87 | | 0.895 | 0.00±6.64 | 0.657 | 0.752 |
| **hs-CRP** | 2.22±10.56 | 0.83±0.79 | 0.289 | -1.02±9.95 | | 0.022 | 0.05±1.11 | 0.651 | 0.206 |

ITT, intention-to-treat; LMCP, low-molecular collagen peptides; WOMAC, Western Ontario and McMaster Universities Osteoarthritis; VAS, visual analog scale; JSW, joint space width; Rt., right; Lt., left; PGA, patient global assessment; ESR, erythrocyte sedimentation rate; hs-CRP, high-sensitive c-reactive protein. **P-*values were compared within each group. ***P*-values were compared between groups. ***joint space width baseline was measured at 90 days.

**Table S3.** Hematological changes between baseline and day 180 among participants in the ITT group

|  | | | | | | | | |  |
| --- | --- | --- | --- | --- | --- | --- | --- | --- | --- |
| **Variable** | **Baseline** | | | |  | | **180 days Change from baseline** | | |
|  | **Placebo (n=40)** | **LMCP (n=40)** | **p**** | **Placebo (n=40)** | | **p*** | **LMCP (n=40)** | **p*** | **p**** |
| **WBC** | 6.09±1.60 | 6.21±1.88 | 0.810 | -0.01±1.60 | | 0.586 | -0.22±0.86 | 0.163 | 0.927 |
| **Hemoglobin** | 13.21±1.20 | 13.58±1.15 | 0.159 | -0.47±0.59 | | <0.001 | -0.60±0.71 | <0.001 | 0.394 |
| **Platelet** | 247.40±38.99 | 266.50±49.58 | 0.059 | 7.38±24.83 | | 0.068 | -10.63±20.35 | 0.001 | <0.001 |
| **AST** | 21.63±7.90 | 21.65±6.22 | 0.707 | -0.85±5.73 | | 0.375 | 0.20±9.05 | 0.603 | 0.992 |
| **ALT** | 19.48±12.08 | 20.13±10.01 | 0.479 | -2.53±10.44 | | 0.125 | -0.08±13.79 | 0.517 | 0.337 |
| **ALP** | 63.68±21.00 | 64.25±20.77 | 0.732 | -0.40±13.11 | | 0.255 | 0.10±10.36 | 0.860 | 0.443 |
| **GGT** | 27.15±40.23 | 23.73±18.04 | 0.751 | -5.28±26.12 | | 0.091 | 1.25±11.36 | 0.877 | 0.209 |
| **Total bilirubin** | 0.47±0.21 | 0.49±0.21 | 0.803 | -0.03±0.21 | | 0.315 | -0.03±0.22 | 0.522 | 0.697 |
| **Total cholesterol** | 205.55±36.22 | 206.78±48.46 | 0.935 | -5.65±28.73 | | 0.221 | 2.80±29.70 | 0.745 | 0.391 |
| **BUN** | 13.97±3.51 | 14.46±4.14 | 0.607 | 0.31±3.36 | | 0.916 | 0.39±4.08 | 0.546 | 0.919 |
| **GFR** | 98.25±10.46 | 95.23±12.02 | 0.164 | 0.85±14.31 | | 0.709 | 0.08±10.61 | 0.844 | 0.729 |
| **Uric acid** | 3.97±0.83 | 4.45±0.90 | 0.026 | 0.28±0.74 | | 0.020 | -0.09±0.52 | 0.420 | 0.011 |
| **Glucose** | 93.55±9.93 | 92.63±11.10 | 0.544 | -0.43±8.16 | | 0.887 | 0.83±16.03 | 0.896 | 0.643 |
| **Creatinine** | 0.69±0.09 | 0.69±0.07 | 0.296 | 0.00±0.08 | | 0.761 | 0.00±0.06 | 0.922 | 0.808 |
| **HDL-c** | 64.03±16.23 | 61.53±12.68 | 0.445 | -3.50±9.01 | | 0.014 | -0.45±8.18 | 0.730 | 0.117 |
| **LDL-c** | 125.15±35.73 | 126.18±46.17 | 0.791 | -6.18±26.33 | | 0.146 | -1.15±26.56 | 0.308 | 0.820 |
| **TG** | 117.05±155.90 | 123.98±76.13 | 0.055 | 6.28±147.34 | | 0.003 | 11.95±73.22 | 0.250 | 0.340 |
| **Na** | 139.55±2.15 | 139.58±2.01 | 0.884 | 0.98±2.11 | | 0.007 | 0.73±2.06 | 0.028 | 0.832 |
| **K** | 4.51±0.23 | 4.35±0.33 | 0.017 | -0.10±0.30 | | 0.041 | -0.04±0.35 | 0.471 | 0.411 |
| **Cl** | 103.05±1.63 | 103.10±1.61 | 0.841 | 0.10±1.72 | | 0.863 | 0.28±1.38 | 0.230 | 0.274 |
| ITT, intention-to-treat; LMCP, low-molecular collagen peptides; WBC, white blood cells; AST, aspartate aminotransferase; ALT, alanine aminotransferase; ALP, alkaline phosphatase; GGT, gamma-glutamyl transferase; TC, total cholesterol; BUN, blood urea nitrogen; GFR, glomerular filtration rate; HDL-c, high-density lipoprotein cholesterol; LDL-c, low-density lipoprotein cholesterol; Na, sodium; K, potassium; Cl, chloride. **P*-values were compared within each group. ***P-*values were compared between groups. | | | | | | | | | |
